# Research on the identification and detection of field pests in the complex background based on the rotation detection algorithm

**DOI:** 10.3389/fpls.2022.1011499

**Published:** 2022-12-13

**Authors:** Wei Zhang, Xulu Xia, Guotao Zhou, Jianming Du, Tianjiao Chen, Zhengyong Zhang, Xiangyang Ma

**Affiliations:** ^1^ Institute of Physical Science and Information Technology, Anhui University, HeFei, China; ^2^ Institute of Intelligent Machines, Hefei Institutes of Physical Science, Chinese Academy of Sciences, Hefei, China; ^3^ Technology Research and Deveplopment Center, Henan Yunfei Technology Development Co. LTD, Henan, China; ^4^ Harvesting and Processing Department, Liaoning Provincial Institiute of Agricultural Mechanization, Shengyang, China

**Keywords:** image recognition, object detection, rotation detection, pest detection, plant protection

## Abstract

As a large agricultural and population country, China’s annual demand for food is significant. The crop yield will be affected by various natural disasters every year, and one of the most important factors affecting crops is the impact of insect pests. The key to solving the problem is to detect, identify and provide feedback in time at the initial stage of the pest. In this paper, according to the pest picture data obtained through the pest detection lamp in the complex natural background and the marking categories of agricultural experts, the pest data set pest rotation detection (PRD21) in different natural environments is constructed. A comparative study of image recognition is carried out through different target detection algorithms. The final experiment proves that the best algorithm for rotation detection improves mean Average Precision by 18.5% compared to the best algorithm for horizontal detection, reaching 78.5%. Regarding Recall, the best rotation detection algorithm runs 94.7%, which is 7.4% higher than horizontal detection. In terms of detection speed, the rotation detection time of a picture is only 0.163s, and the model size is 66.54MB, which can be embedded in mobile devices for fast detection. This experiment proves that rotation detection has a good effect on pests’ detection and recognition rate, which can bring new application value and ideas, provide new methods for plant protection, and improve grain yield.

## 1 Introduction

As the most populous country in the world, China’s annual food demand is the most critical social and livelihood issue. In recent years, urbanization has been getting faster and faster with the rapid development of China’s economy. The immediate problem with it is the reduction of the available agricultural area. In order to ensure that China’s annual grain output can be maintained at 650 billion kg above, it is necessary to improve the efficiency of grain cultivation on limited land. Food production is related to many factors, such as climate, temperature, and humidity (Dayan, 1988). Among them, the most severe threat to food every year is the impact of pests and diseases (Guru-Pirasanna-Pandi et al., 2018). According to the Food and Agriculture Organization of the United Nations statistics, global food production will decrease by 10-16% annually due to the impact of pests and diseases. In China, surveys show that about 40 million tons of food are lost yearly (CCTV News,). The key to solving the problem of grain production is promptly predicting the early formation of pests and scientific control. Therefore, the most critical link is accurately identifying and detecting different pests.

In recent years, traditional machine learning technology has undergone revolutionary changes with the improvement of the computing power of graphics cards and the rapid development of computer software and hardware resources. More and more experts and scholars use their computing power in image recognition. Object detection is a branch of image recognition based on deep learning-based CNN algorithms. At present, CNN has made incredible breakthroughs in theoretical and practical experiments. Current object detection algorithms are divided into two stages and one stage. The main difference is that the second stage forms a series of target candidate boxes and classifies the samples according to the convolutional network; the first stage converts the regression box prediction into a regression problem and then performs regression and sample classification at the same time. The two-stage mainstream target detection algorithms are represented by RCNN (Girshick et al., 2014), Fast RCNN (Girshick, 2015), Faster RCNN (Ren et al., 2017), Cascade RCNN (Cai and Vasconcelos, 2018), and Mask RCNN (He et al., 2017). The mainstream detection algorithms in the first stage are represented by YOLO (Redmon et al., 2016; Redmon and Farhadi, 2017; Redmon and Farhadi, 2018; Bochkovskiy et al., 2020; Ge et al., 2021) series, SSD (Liu et al., 2016), and RetinaNet (Lin et al., 2017).

The development of rotating object detection with horizontal detection has also received more and more attention from researchers. Rotation detection algorithms are represented by R3Det (Yang et al., 2021), ReDet (Han et al., 2021), S2A-Net (Han et al., 2022) and so on. In real environments, most detection objects often appear irregularly, such as text scene recognition in real life (Liao et al., 2018) and ship detection in remote sensing image ports (Fu et al., 2018; Yang et al., 2018; Li et al., 2018). Under these conditions, achieving satisfactory results in horizontal detection is difficult. Based on horizontal detection, rotation detection adds object Angle prediction, which makes the application of rotation detection more extensive. This method can adapt to any Angle and shape transformation of object detection and has good robustness to object localization and classification detection. For example, Ma et al. (2022) used R3Det detection and identification for coastal intensive marine cages. The experimental results showed that the mean Average Precision(mAP) in circular and square cages reached 92.65% and 98.06%, respectively. Peng et al. (2021) applied the rotation detection algorithm to detect insulators in the power grid. The experiments show that R3Det can better determine the position of insulators and reduce economic losses.

Pests live in complex and changeable natural conditions with many species, and the growth patterns of different pests are pretty different. At the same time, some pests are tiny in size and have certain similarities in appearance, color, and other characteristics, making detection and identification difficult. Traditional crop pest detection relies on many experts’ on-site observation, identification, and detection. On the one hand, such detection is time-consuming and labor-intensive. On the other hand, the crops have been seriously affected because many pests can be observed manually, and the best control period is missed. In recent years, the rapid development of target detection algorithms and supporting software and hardware in the field of deep neural network learning has brought the possibility of quick identification and detection of pests, which has extensively promoted the application and development of intelligent plant protection and precision agriculture. Many domestic and foreign scholars conduct computer vision research by processing pest images. For example, M.A. Ebrahimi et al. (2017) proposed to use a machine learning Support Vector Machines(SVM) classifier to detect crops and use SVM to use differential kernel functions to classify and detect greenhouse pests. Li et al. (2021) improved the TPest-RCNN network structure based on the Faster RCNN network. Its backbone uses the VGG16 network for feature learning and uses bilinear interpolation on the candidate coordinates instead of the ROIPool method to generate more accurate values. Finally, classification and coordinate regression correction predictions are performed. Experiments show that whiteflies’ mAP reaches 95% under greenhouse conditions. Cho et al. (2007) collected three pests under greenhouse conditions and proposed using Prewitt for edge detection and counting. Solis-Sánchez et al. (Solis-Sánchez et al., 2011) an improved loss identification algorithm was used to detect six pests under greenhouse conditions.

However, most of the above detection methods mainly classify and identify a single pest image under greenhouse conditions, which has certain limitations in the actual natural environment. The current horizontal target detection network needs more pest training samples to obtain a better recognition rate when training multi-category pests. For example, Liu et al. (2019) An improved convolutional neural network (CNN) and PestNet algorithm with a modular channel attention mechanism were proposed to evaluate 16 pests on 80k datasets MPD2018. The experiment proved that the result of mAP reached 75.46%. The improved convolution network and YOLOv4 network proposed by Tang et al. (2017) integrate attention mechanism and crosses-stage feature fusion to improve feature extraction and fusion capabilities. Experimental results on 28k data and 24 types of pests show that mAP and Recall achieved 71.6% and 83.5%, respectively. Wang et al. (2020) collected data on field pests to obtain 25k pictures with 24 categories and used different level detection algorithms to conduct comparative experiments. Finally, the mAP of YOLOv3 reached 59.37%. The level detection method in the above experiments is used for multi-category experimental research under large-scale data. It can be seen from the above that the horizontal detection method needs extensive data when detecting pests, which takes up many computer resources, and the final detection effect map is only about 75%, which can not reach the practical application value.

**Figure 1:**
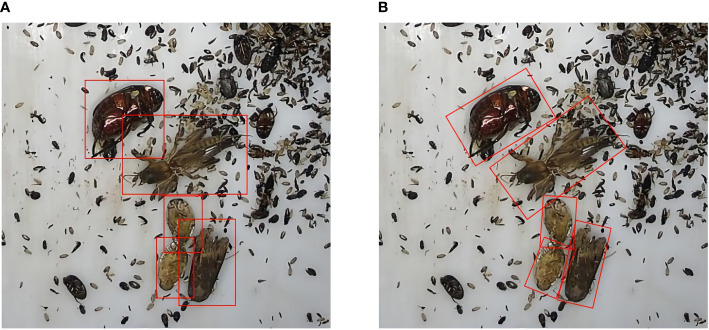
The training samples of horizontal algorithm and rotation detection algorithm are different. **(A)** is the horizontal frame More disturbed by other backgrounds, **(B)** is a rotating frame, which can better fit pest samples.

In this paper, a multi-target pest rotation detection method is proposed. Rotation detection is often used to detect objects with considerable lengths and widths and dense objects, such as ships in remote sensing ports (Fu et al., 2018; Li et al., 2018; Yang et al., 2018). Under the same circumstances, different pests or the same type of pests in motion obtained by the filming equipment will also be affected by different angles, and pests easily pile up densely. Therefore, it is difficult for the horizontal target detection algorithm to achieve a good recognition effect on small and dense targets. As shown in **Figure 1**, the target detection under shade environment level in training will be part of the other characteristics of objects of study, the recognition of samples have larger interference. The rotation detection algorithm can better fit the pest to the samples under the dense shadow, and the performance of the pest can achieve the effect of identifying different poses. This paper will compare the detection differences between different target detection algorithms and rotation detection in different situations to provide a reference for more agricultural pest detection in the future. The main research work of this paper is as follows: (1) Using a variety of horizontal and rotation detection algorithms to detect, identify, compare and analyze field pests. (2) It is concluded that the rotation detection algorithm is generally better than the horizontal detection algorithm in pest detection. The best representative algorithm of rotation detection is selected; (3) In this experiment, a pest rotation detection dataset (PRD21) of 21 pests under the horizontal frame and the rotating frame is constructed, and the difficulty of data detection is classified. It is hoped that the experiment will provide new ideas for accurately identifying pests and diseases and intelligent plant protection, which is conducive to the early and timely detection and prevention of pests and diseases and minimizes economic losses.

## 2 Materials and methods

### 2.1 Introduction to agricultural pests dataset-PRD21

**Figure 2:**
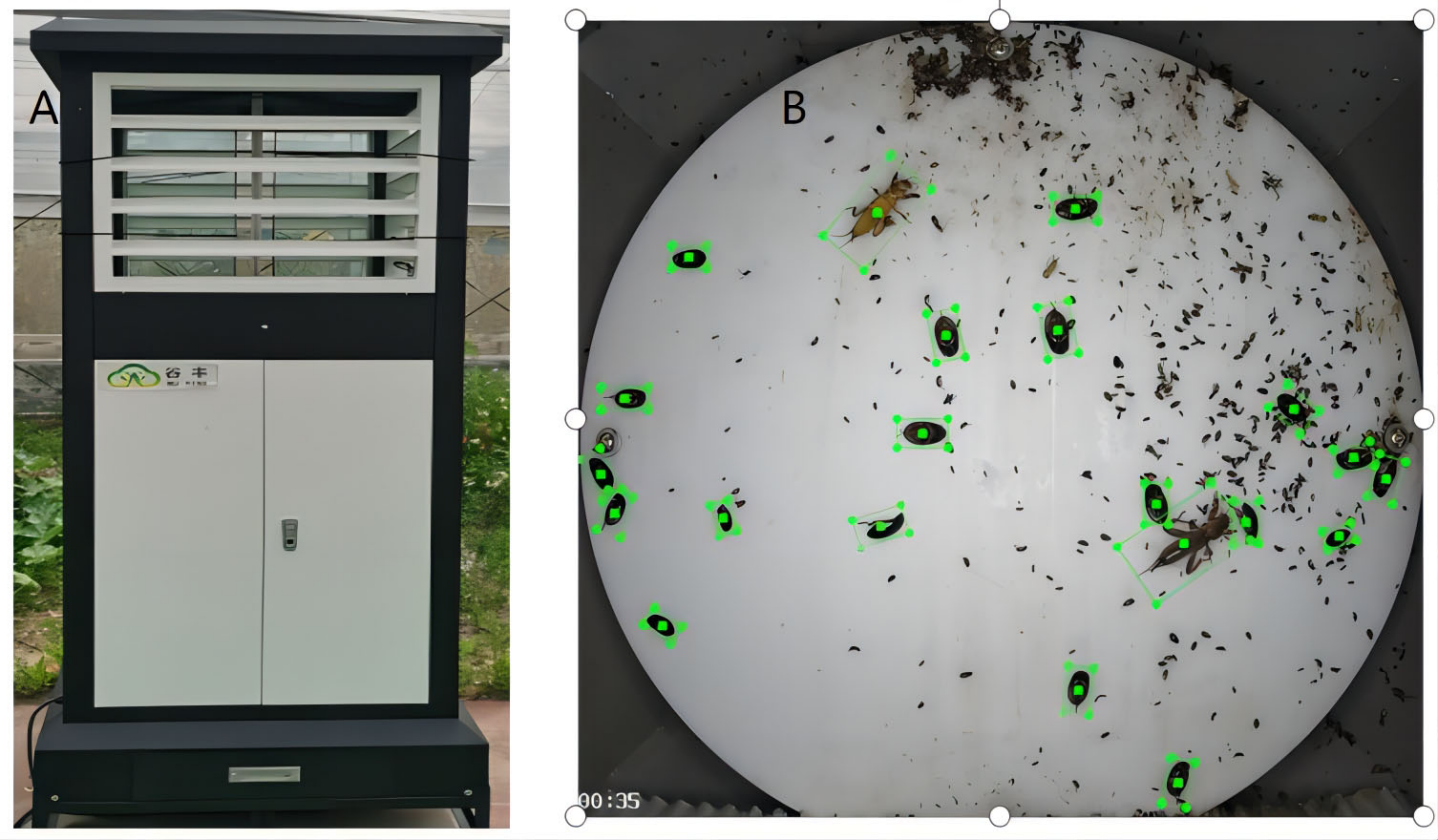
**(A)** is the detection and warning light device for collecting pests. **(B)** shows the collected pest samples.

This experiment ultimately needs to be detected in the natural environment, so the experiment’s data are obtained through the detection and insect detection and reporting trapping equipment to get pest images under natural conditions. As shown in **Figure 2A**, the insect situation monitoring and reporting light device is placed in the actual natural environment to trap pests for 24 hours and automatically set to collect and take photos of pests through the camera in the machine every once in a while and upload them to the background database in time. **Figure 2B** shows the collected pest data samples for a certain period.

A total of 2398 pieces of valuable data were obtained in this dataset, and the image format was unified in JPG format with a resolution of 3840*2160 pixels. According to the pest classification of the Ministry of Agriculture of China and the number of data samples collected in the data set, it is divided into 21 types of pests (Wang et al., 2020). These data are processed into computer-trainable Pascal VOC (Everingham et al., 2010) type data, wherein agricultural experts and lableImg label software generate the training data set for level detection. The rotation detection data is generated by roLabelImg software. Finally, the datasets are divided into 1942 training sets, 216 validation sets, and 240 test sets according to the ratio of 8:1:1.The detected dataset is called Pest Rotate Detection(PRD21).

**Figure 3:**
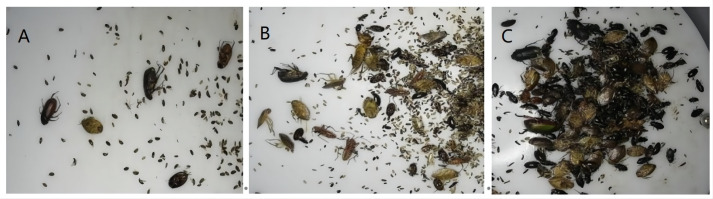
This figure shows the collection of different types of data. **(A)** refers to the occlusion of pests, **(B)** refers to the partial occlusion among pests, and **(C)** refers to the data type with serious occlusion.

**Figure 4:**
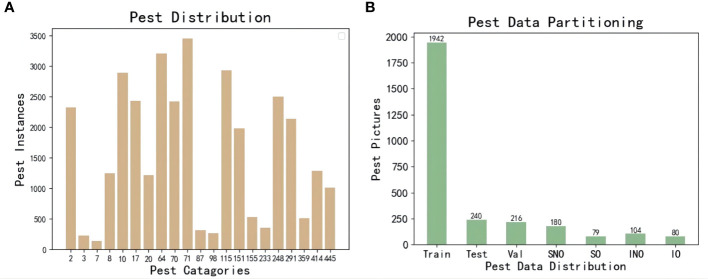
The number of pest instances and data set division. **(A)** is the number of instances in the data set, and **(B)** is the division of the training set.

This paper aims to verify the generalization of the effect of rotation detection in different application scenarios. It is divided by the pest occlusion situation shown in **Figure 3** shows the mutual shielding degree of pests in different environments. **Figure 4** is the name of the specific separated different data sets, namely simple with no occlusion(SNO), simple with occlusion(SO), interference with no occlusion(INO), and interference with occlusion(IO). As shown in **Table 1**, the collected pest species, the pest area, and the relative size of the horizontal frame and the rotating frame are calculated according to Formula (1) and (2). Finally, Formula (3) calculates the severity of occlusion between pests.


(1) $$Ho\text{Re}Scale=\frac{1}{\text{M}}\sum_{\text{M}}^{1}(X_{\max}-X_{\min})*(Y_{\max}-Y_{\min})/C*100\%$$

(2) $$\text{RoReScale}=\frac{1}{\text{M}}\sum_{\text{M}}^{1}(\text{w}*\text{h})/\text{C}*100\%$$

(3) $$\alpha =area(GTBox_{A}\cap GTBox_{B})/area(GTBox_{A}\cup GTBox_{B})$$

**Table 1:** The species of pests and the proportion of relevant sizes.

Index	Pest name	Portrait	Ho Relative scale (%)	Ro Relative scale (%)	Index	Pest name	Portrait	Ho Relative scale (%)	Ro Relative scale (%)
2	Noctuidae	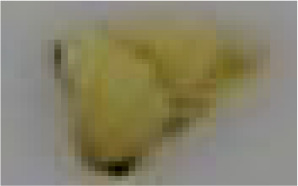	0.206	0.199	98	AnomalaexoletaFald	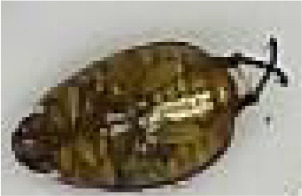	0.131	0.141
3	Athetis Lepigone	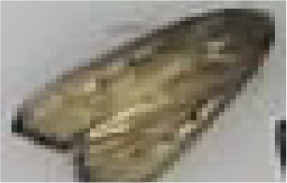	0.194	0.174	115	Diving Beetle	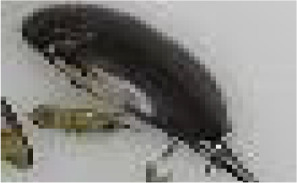	0.133	0.142
7	Spodoptera Litura	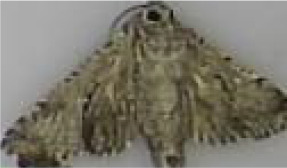	0.14	0.141	151	Cricket	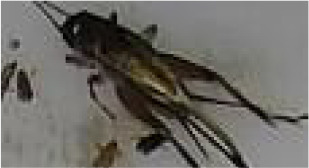	0.236	0.219
8	Mole crickets	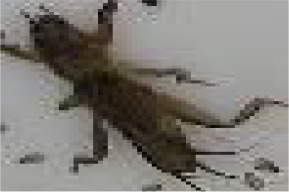	0.697	0.72	155	Sphaerodema Rustica Fabricius	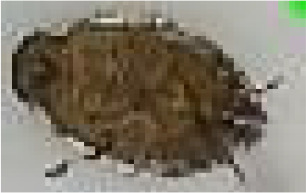	0.155	0.157
10	Snout Moths	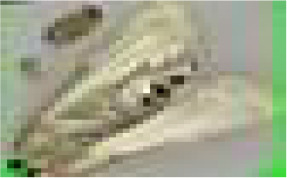	0.117	0.107	233	Spotted Red Bug	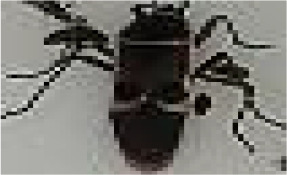	0.132	0.16
17	Helicoverpa Armigera	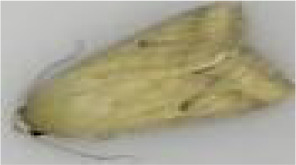	0.209	0.2	248	Marumba Gaschkewitschii	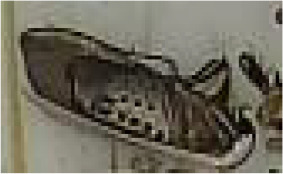	0.998	0.981
20	Oriental Armyworm	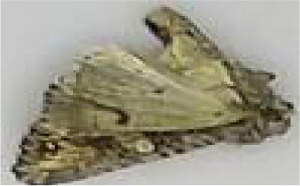	0.196	0.193	291	Carabidae	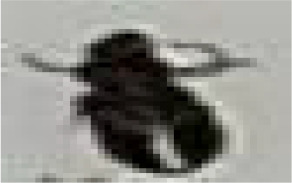	0.084	0.084
64	Holotrichia Parallela	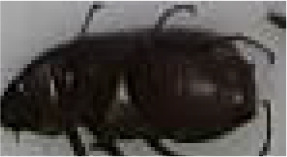	0.192	0.194	359	Cockchafer	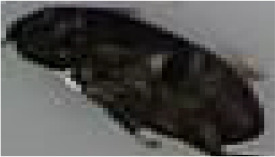	0.13	0.134
70	Anomala corpulenta Motschulsky	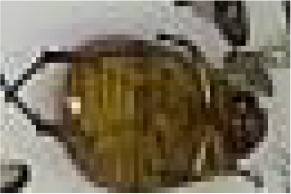	0.264	0.284	414	Turtle Shell	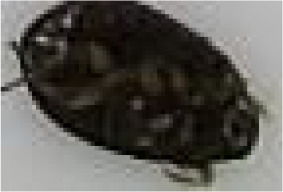	0.092	0.084
71	Coleopters	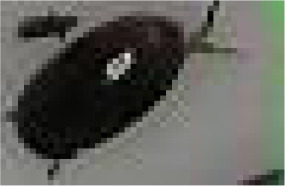	0.099	0.097	445	Metaboluo Impressifros Fairmaire	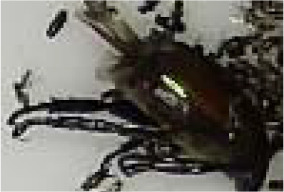	0.136	0.161
87	Tiger Beetle	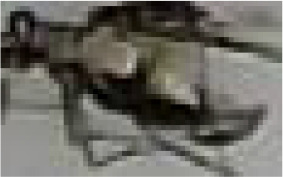	0.085	0.083					

Formula 1 is the area and relative proportion of the horizontal frame, and Formula 2 is the area and relative proportion of the rotating frame. C is the image’s original size, and M is the total number of instances of a specific class. X_i_ is the horizontal relative position value of the corresponding pest, and Y_i_ is the vertical value of the corresponding pest. w and h are the width and height of corresponding pest coordinates. The function area() represents the area of the two pest objects,s A and B, ∩ where the two pest objects intersect and ∪ where the two pest objects are combined. α is the scaling factor, and its value is between 0 and 0.2. When α>0.1, it was considered that the two pests had severe shading; when α<0.1, it was supposed to be slightly shading. GTBox is the area of a single pest.

### 2.2 The algorithm model used is introduced

This experiment uses the horizontal box target detection one-stage algorithms RetinaNet, YOLOX, YOLOv5, YOLOv6, and two-stage algorithms Faster RCNN and Cascade RCNN for comparison experiments. Rotation detection includes ReDet, R3Det, Rotated Faster RCNN, and S2ANet as comparison algorithm models.

#### 2.2.1 Introduction to algorithm models related to horizontal object detection

##### 2.2.1.1 Faster RCNN introduction

This algorithm is an improved and optimized classic CNN convolution network algorithm. First, use the convolution layers for feature extraction to obtain feature maps and generate region proposals through Region Proposal Networks. The region of interest in Roi Pooling is extracted through feature maps and proposals, and the accurate location and category of the detection target are finally determined through the fully connected layer and bounding box regression.

##### 2.2.1.2 Cascade RCNN introduction

This algorithm further optimizes the threshold setting in Faster RCNN, cascades multiple regressors and detectors with different thresholds, and continuously improves the threshold multi-cascade network structure iteratively. Ultimately, the accuracy of detecting target locations is maximized.

##### 2.2.1.3 YOLOX introduction

As a single-stage target detection algorithm of the You Only Look Once(YOLO) series, positioning and classification are performed simultaneously. The generation method of anchor free is adopted to reduce the amount of calculation. The network structure mainly includes four parts, 1) Input: input image and perform data enhancement. 2) Backbone network (CSPDarknet53 (Wang et al., 2020)): Mainly used for feature extraction. 3) Neck: This layer uses Feature Pyramid Network(FPN) (Lin et al., 2017) and Path Aggregation Network(PAN) (Liu et al., 2018) as feature fusion. 4) Head: This layer predicts classification and location results.

##### 2.2.1.4 YOLOv5 introduction

The network structure of the algorithm can be divided into four parts, the Input layer, the Backbone network, the Neck network, and the Prediction layer. The backbone network consists of Focus, CSP, and Spatial Pyramid Pooling module layers (Zhang et al., 2022). The Neck layer uses the residual network to improve the feature fusion ability. In the prediction layer, the loss of the regression box is calculated by GIoU Loss (Rezatofighi et al., 2019), and three different scale predictions are obtained, divided into 80×80, 40×40, and 20×20. The BCELogitsLoss function calculated Objectness-loss and Classification-loss. Finally, the best prediction results are selected according to three dimensions.

##### 2.2.1.5 YOLOv6 introduction

As the latest algorithm of the YOLO series, many algorithm improvements have been made. Initially, the anchor-free method was used to generate the prediction frame and the same data enhancement as YOLOv5. The backbone network uses EfficientRep to replace the previous CSPDarknet for feature extraction. Neck built Rep-PAN based on Rep and PAN for feature fusion. The Head layer is decoupled in the same way as YOLOX, which separates the efficient structure of regression and category classification. The label assignment selection uses simOTA (Ge et al., 2021) to equalize the positive and negative samples. Finally, a new regression loss SIOU (Gevorgyan, 2022) is introduced to reduce the degree of freedom of regression to accelerate network convergence and further improve the accuracy of regression. From the above, we can be found that YOLOv6 combines the advantages of YOLOv5 and YOLOX.

##### 2.2.1.6 RetinaNet introduction

As a one-stage target detection algorithm, the network structure is backbone using (vgg, resnet) for feature extraction, and then through Feature Pyramid Networks(FPN) to enhance the feature map of target area information for features of different scales, and finally predict the target frame in two FCN layers location and category. The main innovation of this structure is that Focal Loss is added to the one-stage detector to optimize the sample category imbalance problem, and anchor boxes are used to generate prediction boxes.

#### 2.2.2 Introduction to algorithm models related to rotating target detection

##### 2.2.2.1 ReDet introduction

When the traditional convolution network detects objects in any direction, it usually enhances the rotation data in the training samples, so the detection effect is poor, and more inclined models are required. The ReDet algorithm uses the equivariant rotation network combined with the detector to obtain the rotation features, uses the rotation invariant RiRoi Align space and the angle dimension to extract the features, and finally predicts the output.

##### 2.2.2.2 S2ANet introduction

Due to the rotation detection network’s rotation characteristics, sometimes the generated anchor box has a high degree of confidence, but there is still a significant dislocation in the instance fitting. To optimize this problem, S2A-Net adopts RetinaNet (Lin et al., 2017) as the backbone, plus FPN and component Feature Alignment Module (FAM) (Wang et al., 2019) and Oriented Detection Module (ODM) (Xie et al., 2021) modules for region selection and feature extraction fusion.

##### 2.2.2.3 R3Det introduction

**Figure 5:**
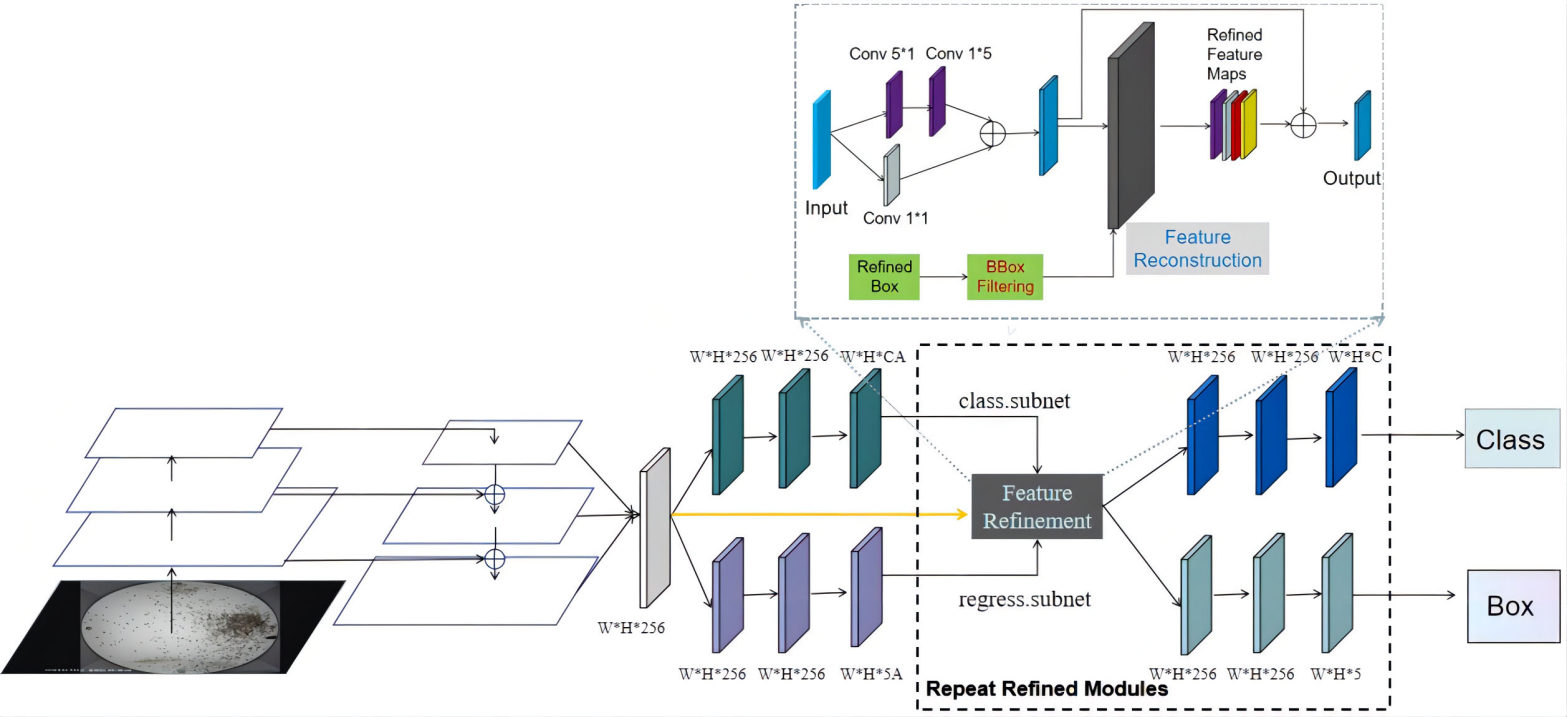
The network structure diagram of the rotation detection algorithm used in this experiment.


This experiment uses the R3Det rotation detection algorithm as a research method to compare other horizontal detection and rotation detection. The network structure is shown in **Figure 5**. The algorithm designed a refined one-stage accurate and a fast detector that combined the anchor points of the horizontal target detection algorithm and the anchor points of the rotation detection algorithm. The final effect significantly improved the adaptability of pest recognition in different scenes. Firstly, horizontal detection anchors are used to generate more candidate regions. Secondly, rotating anchors are used to optimize the dense target scene further. In the middle, the feature refinement module (FRM) (Yang et al., 2021) is used to refine and accurately process the predicted target locations. In order to achieve feature alignment, the algorithm uses Range non-maximum Suppression(RNMS) (Yang et al., 2021) instead of traditional non-maximum Suppression(NMS) (Neubeck and Van Gool, 2006). This part of the improvement method sets different filtering thresholds according to the number of samples and appearance characteristics of different pest categories. In terms of the loss function, the algorithm uses the approximate SkewIoU loss function, which can be pushed to calculate the multi-objective and multi-task rotation box. Further, it optimizes the problem of difficult identification of small objects and sample imbalance. The relevant calculation formulas are shown in the following (4-6).


(4) $$SkewIoU=\frac{area(c1\cap c2)}{area(c1\cup c2)}$$

(5) $$L_{loss}=\frac{\lambda_{1}}{S}\sum_{s=1}^{S}obj_{s}\frac{L_{reg}({v^{\prime }}_{n},v_{n})}{\left|L_{reg}({v^{\prime }}_{n},v_{n})\right|}|f(SkewIoU)|+\frac{\lambda_{2}}{S}\sum_{s=1}^{S}L_{cls}(p_{s},t_{s})$$

(6) $$L_{reg}(v^{\prime },v)=L_{smooth-l1}({v^{\prime }}_{\theta },v_{\theta })-IoU({v^{\prime }}_{\{x,y,w,h\},}v_{\left\{x,y,w,h\right\}})$$

Where S is the number of anchor boxes when the parameter obj is 1, it means the foreground, and when it is 0, it means the background. v’ and v represent the ground-truth box’s prediction vector and target vector. p_n_ is the probability distribution of various types, and t_n_ is the corresponding target label. SkewIoU is the overlapping area of the predicted and ground-truth boxes. λ is the sum of different weights and is 1. Finally, f(SkewIoU) and L_reg_ are combined as the regression gradient function.

### 2.3 Evaluation indicators

The evaluation criteria used in this experiment are single-class Average Precision (AP), single-class Recall, all-class average precision mAP, all-class average recall rate mean Average Recall (mR), model parameters, and detection time comparisons analysis. The relevant calculation formula is shown in the following (7-10).


(7) $$P=\frac{TP}{TP+FP}\times 100\%$$

(8) $$R=\frac{TP}{TP+FN}\times 100\%$$

(9) $$AP=\int_{0}^{1}P(R)dR$$

(10) $$mAP=\frac{1}{M}\sum_{k=1}^{M}AP(k)\times 100\%$$

Where TP and FN are the numbers of positive and negative samples predicted to be positive, FP is the number of negative samples predicted to be positive, and M is the total number of classes in the data. P is precision, R recalls, and AP is precision for a single class.

## 3 Experimental

### 3.1 Experimental environment

The operating platform of this experiment is the Ubuntu20.04.4 system. The CPU is Intel Core i9-9900K, the frequency is 3.6GHz, and the running memory is 16G. The graphics card is NVIDIA TITAN RTX, and the GPU memory is 24G. The CUDA version is 10.2, and the CUDNN accelerated version is 7.6.5. PyCharm Professional Edition, Python 3.7.11 interpreter, MMCV version 1.4.0, and Pytorch 1.10 deep learning framework are used.

### 3.2 Experimental procedure

In the experiment, under the same training set, the number of iterations epoch is 36, the batch size is 4, the learning rate is 0.01, and the value is dynamically optimized during the training process. Momentum is 0.9, and weight decay is set to 0.0005. SGD is a parameter optimizer to train and validate different classification test datasets.

#### 3.2.1 Comprehensive comparison between rotation detection and horizontal detection algorithms

In this experiment, the most representative horizontal detection algorithms and rotation detection algorithms are selected as comparisons. Some of them have the same backbone network structure and are adjusted to Resnet101, and the input image size is scaled to (1800, 1200) during training. During the test, experimental verification was carried out in 5 different scenarios, and the experimental results are shown in **Table 2**.

**Table 2:** Comprehensive model comparison results.

Algorithm model	Backbone	Test240%		SNO180%		SO79%		INO104%		IO80%	
	Indicators	mAP	mR	mAP	mR	mAP	mR	mAP	mR	mAP	mR
two-stage											
Faster RCNN	Resnet101	53.6	85.3	56.1	84.1	62.0	82.5	56.8	84.3	44.7	68.2
Cascade RCNN	Resnet101	52.3	83.2	54.9	83.4	58.2	77.2	53.6	77.6	45.9	70.2
one-stage											
RetinaNet	Resnet101	46.1	87.3	50.6	94.3	48.0	80.3	47.6	88.1	34.0	72.3
YOLOX	CSPDarknet	57.0	82.0	61.3	82.6	65.5	78.2	53.4	72.2	47.8	75.6
YOLOv5	CSPDarknet	60.0	63.0	62.5	57.3	65.9	67.4	62.2	61.8	53.4	57.0
YOLOv6	EfficientRep	58.2	54.7	60.5	54.3	66.4	51.4	64.6	53.0	50.8	52.3
rotation detection											
RoFaster RCNN	Resnet101	59.3	88.8	58.6	81.2	69.1	81.4	65.3	86.8	53.6	82.0
ReDet	Resnet101	54.4	87.9	54.6	87.2	60.3	77.7	51.6	83.4	43.4	73.5
S2ANet	Resnet101	60.2	**94.7**	60.0	95.7	69.0	**92.2**	63.8	90.6	54.2	**93.4**
R3Det	Resnet101	**78.5**	93.6	**85.1**	**99.1**	**82.6**	89.7	**79.0**	**91.9**	**70.3**	85.0

The bold numbers in the table indicate the highest values of he experimental results.

It can be seen from the experimental results that the YOLO series algorithm is better than other detection algorithms in mAP. The best level detection algorithm is the YOLOv5 model, which is 6.4%, 7.7%, 3%, and 13.9% higher than Faster RCNN, Cascade RCNN, YOLOX, and RetinaNet at mAP0.5. Regarding recall rate, YOLOv5 and YOLOv6 in the YOLO series are far lower than other detection algorithms, only YOLOX can reach more than 82%, and the algorithm with the highest recall rate for horizontal detection is RetinaNet, which reaches 87.3%. The experiments show that both the one-stage and two-stage target detection algorithms have advantages and disadvantages. Compared with the rotation detection algorithm, the best one-stage algorithm is far lower than the RoFaster RCNN, R3Det, and S2ANet algorithms. RoFaster RCNN is 5.7% and 3.5% higher than Faster RCNN in mAP and Recall under the same conditions. On the same Backbone, R3Det is 24.9%, 26.2%, and 32.4% higher than Faster, Cascade, and RetinaNet algorithms.

#### 3.2.2 Influence of backbone network and image input size

As seen above, rotation detection has initially demonstrated its advantages. In practice, many factors affect the final result of different algorithms. For example, the backbone network and the input image size play a crucial role in the feature extraction of the target object. This paper conducts comparative research experiments on these two effects in different scenarios. The same backbone network is still set to Resnet101, the YOLOv5 and YOLOv6 use CSPDarknet and EfficientRep as the backbone network, respectively, and the image input size during training and testing is adjusted to (1000, 600). The experimental results are shown in **Table 3**.

**Table 3:** Comparison of detection results when the input image is 1000*600.

Algorithm model	Backbone	Test240%		SNO180%		SO79%		INO104%		IO80%	
		Indicators	mAP	mR	mAP	mR	mAP	mR	mAP	mR	mAP	mR
Faster RCNN	Resnet101	49.2	76.2	50.7	76.8	55.9	72.1	52.1	78.2	42.5	74.3
Cascade RCNN	Resnet101	48.7	69.8	51.0	74.1	54.8	70.0	49.3	65.2	41.2	67.3
YOLOv5	CSPDarknet	55.8	59.7	62.3	61.1	65.4	67.4	59.4	53.5	50.1	57.0
YOLOv6	EfficientRep	57.2	51.9	60.4	58.7	64.8	54.3	58.1	54.1	47.6	54.3
RoFaster RCNN	Resnet101	57.2	83.0	55.0	87.1	67.7	82.2	63.9	84.7	49.9	80.8
ReDet	Resnet101	44.8	76.0	51.3	85.3	53.6	74.6	46.1	74.4	37.5	66.0
S2ANet	Resnet101	56.5	**93.6**	57.6	95.2	64.9	**89.2**	60.2	90.2	47.4	**90.6**
R3Det	Resnet101	**70.6**	91.9	**78.6**	**97.7**	**74.8**	85.8	**77.9**	**91.4**	**55.1**	76.8

The bold numbers in the table indicate the highest values of the experimental results.

Through the comparison of experimental results, it is found that each algorithm has a certain degree of reduction when the input size is reduced. When the size is reduced, YOLOv5 and YOLOv6 mAP drop by 4.2% and 1%, respectively, under Test240. Other horizontal detection Faster RCNN and Cascade RCNN algorithms reduce mAP by 4.4% and 3.6% and Recall by 9.1% and 13.4%, respectively. The rotation detection algorithm declines further; the minor reduction is 2.1% of RoFaster RCNN, and the most significant drop is 7.9% of R3Det. Experimental results show that the image size change substantially impacts the final result. Except for the ReDet algorithm, other rotation detection algorithms are still better than the horizontal detection algorithm model. To verify the influence of the backbone network of the algorithm, continue to join the experiment. Keep the training image input size as (1800,1200) while setting the backbone adjustment depth to Resnet50. The experimental results are shown in **Table 4** below.

**Table 4:** Comparison of detection results when the input picture is 1800*1200.

Algorithm model	Backbone	Test240%		SNO180%		SO79%		INO104%		IO80%	
		Indicators	mAP	mR	mAP	mR	mAP	mR	mAP	mR	mAP	mR
Faster RCNN	Resnet50	52.5	82.5	53.8	74.5	59.3	77.9	57.7	67.7	45.2	66.1
Cascade RCNN	Resnet50	53.1	75.6	56.2	86.3	58.6	71.4	53.2	84.4	43.3	77.1
RoFaster RCNN	Resnet50	58.3	85.6	59.0	87.2	68.1	87.4	64.1	86.3	52.9	83.2
ReDet	Resnet50	52.7	87.0	55.0	86.3	59.9	78.6	53.3	85.7	41.6	69.7
S2ANet	Resnet50	58.8	**95.6**	59.2	97.2	68.4	**91.6**	63.5	**92.2**	51.7	**94.7**
R3Det	Resnet50	**77.7**	94.0	**76.1**	**98.2**	**72.9**	87.8	**74.0**	91.5	**59.0**	79.1

The bold numbers in the table indicate the highest values of the experimental results.

We can be seen from the results that when the image training size is (1800, 1200) and the backbone network depth is reduced to Resnet50, the horizontal detection and rotation detection algorithms have a slight reduction. Among them, the algorithm with the most negligible reduction is 0.8% of R3Det, and the highest is only 1.7%. The highest reduction of the horizontal detection algorithm above the recall rate is 7.6% of Cascade RCNN, and the rotation detection algorithm has almost no change. However, experiments show that when the data size is large, the network training model has less influence on the depth of feature extraction.

#### 3.2.3 Analysis of recall and mAP of different algorithms in different types of datasets

**Figure 6:**
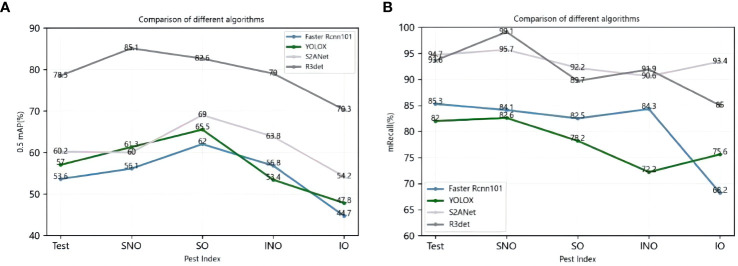
Left panel **(A)** shows the mAP of the four algorithms on the Test datasets, and correct panel **(B)** shows the Recall of the corresponding algorithms and datasets.

This experiment selects four algorithms with the best detection effect for comparison. The horizontal one-stage detection algorithm is YOLOX, the second-stage detection algorithm is Faster RCNN, and the rotation detection algorithm is R3Det and S2ANet. Take Test240 data as the test set for the model. The comparison of mAP and mean Average Recall(mRecall) is shown in **Figure 6**.

**Figure 6** shows that at mAP, S2ANet is higher than other level detection algorithms for various pests under different environmental conditions, and the mAP is only lower than 1.3% on SNO. The detection effect of R3Det in different test sets, mAP reached 78.5%, 85.1%, 82.6%, 79%, and 70.3%, respectively; this shows that R3Det is more efficient and flexible in the detection of dense target pests through the refinement module and the feature reconstruction module.

In the mRecall comparison, although the mAP of YOLOX is higher than that of Faster RCNN, the recall rate is lower than that of Faster RCNN. The two algorithmic models of rotation detection outperformed the horizontal detection algorithm. Rotation detection achieves the highest Recall of more than 95% on the SNO simple data set. The Recall calculated by R3Det is above 86% on all types of data sets, which shows that the horizontal anchor frame and the rotation frame used by R3Det are combined to improve the recall rate. At the same time, the approximate SkewIoU loss function is used to achieve more accurate rotation. Finally, the results show that the recall rate can be significantly improved, which has good results under austere conditions and overcomes the problem of dense scenes.

In summary, whether a one-stage or two-stage target detection algorithm, the detection effect is not as good as rotation detection in various environments. In contrast, other rotation algorithms, such as S2ANet and RoFaster RCNN, have an excellent recognition ratio. In particular, the R3Det algorithm still performs well in environments with severe occlusion and more complex backgrounds, which shows that the rotation algorithm has good results in remote sensing data and a reasonable recognition rate in pest detection in different fields in the field and generalization rate.

#### 3.2.4 Analysis of a single type of pest

**Figure 7:**
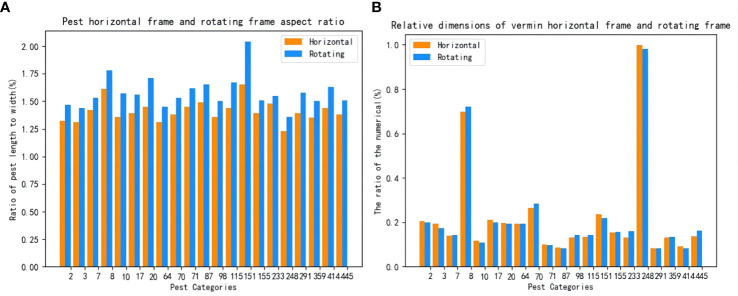
Left panel **(A)** shows the aspect ratio data of A single class of pests, and right panel **(B)** shows the proportion of the relative original size of A single type of pest.

The total categories of the data set in this experiment are 21 categories. The growth shape and other characteristics of different pest types have specific differences, and some attributes of some categories are similar. In order to provide a theoretical reference for identifying more varieties of pests in the future, this paper analyzes the influence of characteristics of different pests. **Figure 7** shows the aspect ratio and relative size of a single category of pests. The algorithm model is trained with horizontal detection and rotation detection. The single-category AP50 of different algorithms is calculated, and the results are shown in **Table 5**.

**Table 5:** AP50 for a single class.

Method	2	3	7	8	10	17	20	64	70	71	87	98	115	151	155	233	248	291	359	414	445
Faster RCNN	32.4	37.3	32.9	74.3	28.8	59.4	61.1	54.8	81.6	60.8	54.7	60.1	47.4	68.0	58.7	26.4	88.1	17.7	53.3	42.4	85.3
Cascade RCNN	30.0	34.0	15.3	77.0	24.8	44.8	61.8	63.1	77.7	61.4	50.2	44.9	40.9	68.1	53.3	55.5	**92.7**	18.5	68.6	41.1	75.5
RetinaNet	28.2	29.1	6.6	73.4	29.6	48.2	47.8	53.2	76.1	58.2	51.7	47.1	30.4	74.0	54.1	41.2	59.5	14.7	58.0	27.1	59.6
YOLOX	47.5	37.9	**56.8**	77.2	40.4	49.0	54.5	56.5	76.0	63.7	26.9	63.0	44.8	59.5	57.2	69.1	70.0	23.6	74.8	60.3	87.4
YOLOv5	32.3	26.5	33.0	71.1	35.4	49.2	52.1	52.6	80.7	64.4	50.0	71.6	35.8	63.6	58.1	71.9	81.9	21.4	72.6	55.5	86.3
RoFaster RCNN	27.9	46.3	36.4	80.2	36.7	64.4	56.9	63.6	78.4	66.3	62.5	62.3	52.5	75.4	66.3	46.8	85.5	25.0	74.3	54.4	82.8
ReDet	27.0	43.5	14.1	80.4	28.7	53.2	52.2	55.2	75.4	60.2	53.7	51.7	42.6	68.3	57.1	46.9	85.7	20.5	62.0	50.5	76.7
S2ANet	28.0	35.2	29.8	85.7	39.6	65.1	58.1	61.4	83.8	71.2	58.2	61.6	57.0	76.5	70.9	53.1	90.7	23.4	70.7	62.6	80.6
R3Det	**76.5**	**55.6**	14.7	**89.7**	**77.8**	**86.0**	**89.6**	**73.0**	**87.1**	**85.7**	**79.7**	**88.3**	**83.0**	**90.0**	**90.4**	**72.7**	81.8	**62.8**	**96.3**	**69.7**	**97.9**

The bold numbers in the table indicate the highest values of the experimental results.

It can be seen from **Table 5** that under the same data conditions, the aspect ratio of the rotating frame is larger than the scale of the horizontal structure, and the relative proportion of the rotating frame is lower than that of the horizontal frame. In general, the area occupied by pests is small. It shows that the detection and recognition of tiny pests are complex, and the training samples of the rotating frame can better fit the target object. The interference of other environmental factors on the models during training in different scenarios is also reduced. Therefore, the rotation detection algorithm can still achieve good results under more complex or denser conditions.

The table shows the single-class experimental results for the selected model comparisons. It can be concluded from this table that when the aspect ratio of pests is greater than 2, only one pest is the 151st pest, and the mAP of this pest is 90%. When the ratio is [1.75, 2), the mAP of the 8th class of pests is 89.7%. When the ratio was [1.65, 1.75], including the 87th, 20th, and 115th types of pests, the mAP was 79.7%, 89.6%, and 83%, respectively. When the ratio was [1.55, 1.65), there were 6 species of pests; the highest was 86% of class 233, and the lowest was 62.8% of class 291. There are also six classes where the ratio is [1.50, 1.55), where the best detections are 97.9% for class 445 and 96.3% for class 359. When the ratio was lower than 1.5, there were four classes, 2, 248, 3, and 64, with mAP of 76.5%, 81.8%, 55.6%, and 73%, respectively.

After analysis, there were 15 types of detected pests with aspect ratios between [1.5, 1.75], accounting for 71.4% of the total detected pest species. The R3det rotation detection algorithm is generally more effective than other horizontal detection algorithms in detecting these categories. When it is lower than 1.5, the rotation detection still performs well. Experiments show that the rotation algorithm detection not only has a good effect on detecting pests at a high aspect ratio but also has a reasonable recognition rate when the ratio is low. For example, in comparing 21 categories of total pests, R3det is the highest in 19 pests, second only to Cascade RCNN in the 248th category of pests, but still achieves an mAP of 81.8%. The analysis results further demonstrate that the R3det model can detect most pests.

#### 3.2.5 Comparative analysis of detection speed and parameter quantity

Regarding recognition rate, the rotation detection algorithm has shown better results than the horizontal detection. However, timely detection of changes before and after pests and diseases and making correct judgments are the key to agricultural control. Therefore, the detection time is also an important indicator. On the other hand, different detection algorithm models finally need to be transplanted to specific hardware devices for mobile deployment. However, due to the limited resources of various hardware devices, they cannot carry large capacities; Therefore, the model’s size is also one of the essential considerations when choosing a suitable algorithm. Finally, as shown in **Table 6**, we compared the model parameters and detection time of different models under different backbone network depth conditions and when the image input size changes during training.

**Table 6:** Comparison of detection speed and parameter amount of the same backbone network algorithm.

Algorithmmodel	Backbone Resnet 50 1800*1200(1800*1200)			Backbone Resnet 101 1000*600(1000*600)			Backbone Resnet 101 1800*1200(1800*1200)		
	FPS	Single graph detection time/s	GFLOPs/MB	FPS	Single graph detection time/s	GFLOPs/MB	FPS	Single graph detection time/s	GFLOPs/MB
Faster RCNN	13.5	**0.074**	41.23	25.3	**0.040**	60.22	10.1	**0.100**	60.22
Cascade RCNN	12.0	0.083	68.99	21.1	0.047	87.98	9.2	0.109	87.98
RoFaster RCNN	12.5	0.08	41.14	22.1	0.045	60.14	9.5	0.105	60.14
ReDet	8.8	0.114	40.23	16.8	0.060	58.22	6.7	0.149	58.22
S2ANet	10.8	0.092	**38.63**	20.8	0.048	**57.62**	8.4	0.119	**57.62**
R3Det	7.2	0.138	47.54	14.0	0.071	66.54	6.1	0.163	66.54

The bold numbers in the table indicate the highest values of the experimental results.

It can be seen from the experimental results that on the same backbone network, the rotation detection algorithm is slightly lower than the horizontal detection algorithm in the detection speed of a single image. The maximum time of the rotation detection algorithm for a single image is only 0.163s, which can meet the requirements of practical detection applications. Similarly, in terms of the number of algorithm models, the parameters of RoFaster, ReDet, and S2ANet algorithms are all lower than those of the horizontal detection algorithm. The performance of R3Det is slightly higher than that of the horizontal detection algorithm, but the amount of parameters is only 66.54MB. The practice has proved that the algorithm can be flexibly applied to the embedded mobile deployment of pest-monitoring lights.

#### 3.2.6 Pest detection visualization comparison

**Figure 8:**
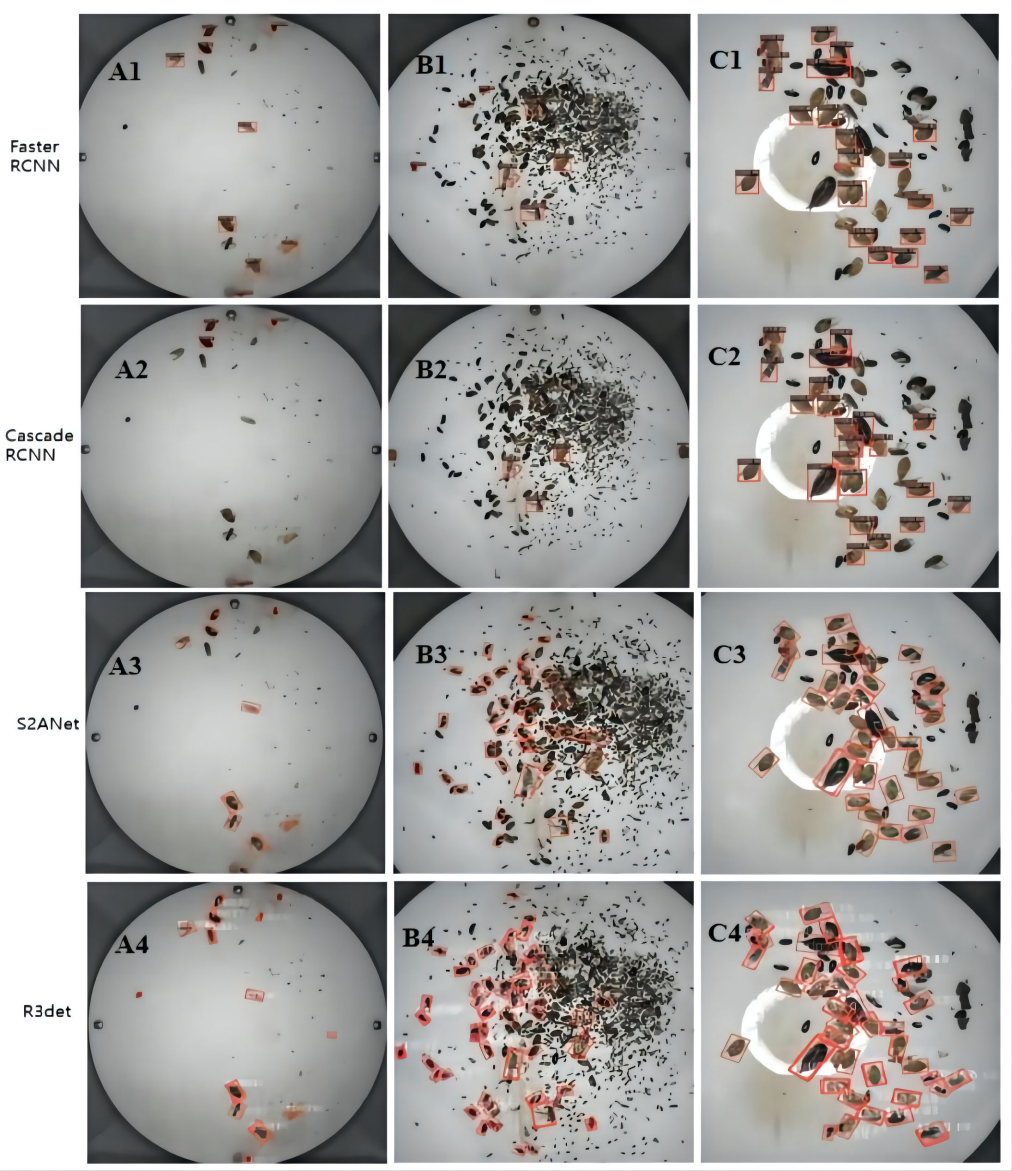
Comparison between horizontal algorithm and rotation algorithm. The algorithm model for comparison is Faster RCNN, Cascade RCNN, S2ANet and R3Det. Test figure **(A)** represents small-target pest detection, **(B)** represents intensive pest target detection, and **(C)** represents interpest occlusion type detection.

**Figure 9:**
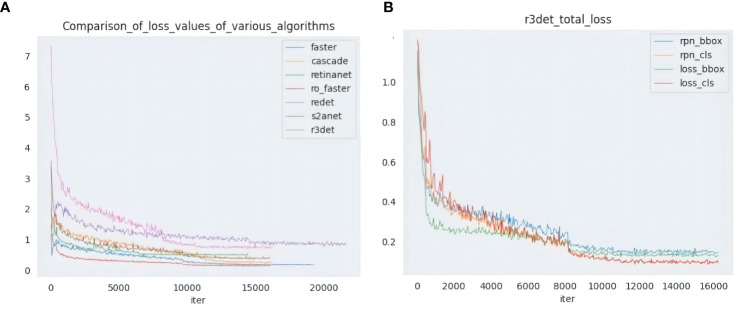
Left panel **(A)** shows the Loss comparison of multiple algorithms, and right panel **(B)** shows multiple Loss curves of the R3Det algorithm.

Through the above comparative studies in different aspects, it is found that rotation detection algorithms such as R3det have better detection results. In this experiment, to verify the detection effect in the actual scene, the Faster RCNN and Cascade RCNN with the best horizontal detection effect were selected, and the rotation detection was compared with R3det and S2ANet as the representative algorithms. The threshold was set to 0.5, and the test data included small targets, dense and occlusion type 3, the detection effect is shown in **Figure 8**, and **Figure 9** compares the decreasing trend of the loss of different algorithms.

It can be seen from the comparison effect that R3det can detect all pests in small target detection. The detection results of Faster RCNN and S2ANet are the same. Meanwhile, Cascade RCNN has the worst detection performance, only detecting a few pests. In dense scenarios, the horizontal detection algorithm can only detect a few pests, which is far from meeting the actual needs. The rotation detection algorithm shows its superior detection ability in a dense environment. And the detection capability is much higher than horizontal detection, and more pests can be detected in this environment. In practical situations, pests are prone to occlusion when they appear in piles. The horizontal detection algorithm is prone to be disturbed by other target features during training and has a seriously missed detection rate. In this case, rotation detection can better fit the pest samples under different postures and accurately identify the blocked pests. Among them, the R3det algorithm can account for both small targets and occluded pests in the case of occlusion.

## 4 Discussion and conclusion

Detecting agricultural pests has always been a complex problem for many experts and scholars. Insect pests will not only eventually reduce crop yield but also may impact the ecological balance of a specific area. Therefore, accurate identification and detection of pests in complex scenarios is the key to the environmental protection of crops. Traditional reliance on agricultural experts for on-site inspection and testing is inefficient and time-sensitive, often missing the optimal period of protection. In the current research on deep learning object detection, it is found that horizontal detection has a certain effect on the simple background of a single pest. However, the product is difficult to meet the actual requirements in complex multi-category environments. In this paper, the rotation detection algorithm is firstly proposed to be applied to the pest detection field of the constructed pest datasets PRD21, and good detection results have been achieved, which provides a new solution for pest detection in the early stage of agriculture. Among them, the R3Det algorithm uses its refinement module to improve the recognition rate and approximate SkewIoU loss to improve the recall rate. Finally, the detection comparison in the actual environment proves its superiority and strong adaptability. The overall experimental conclusions are as follows:

1) This paper uses rotation and horizontal detection algorithms to research pest detection and identification. Under different natural image detection environments, rotation detection reflects the advantages of good generalization and strong adaptability. The R3det algorithm can still achieve a recognition rate of more than 70% under more occlusion and serious background interference, and the Recall also reaches 86.0%. It achieves 85.1%, 82.6 and 79% under the other classification test data sets, SNO, SO, and INO.

2) In single-class detection, the performance of rotation detection is the highest in 19 of the 21 categories. The highest category is the 445th category, which reaches 99.7%, and the other category achieves 81.1%. The detection effect shows that the rotation algorithm has good robustness to multi-category targets in addition to the influence of environmental factors.

3)Since pests may increase over time over large areas, it is necessary to detect and identify pests in the exact location within a short period. Through experiments, it has been found that the detection time of a single image of the rotation detection algorithm is less than 0.17s, which can realize rapid identification and detection.

The above experiments prove that rotation detection has practical application value on pests. However, at the same time, there are some deficiencies. For example, the detection effect of category 7 pests is low, and there is still room for improvement when the environment is the most complex. In the future, we will further collect samples of various pests in different environments and add specific pest categories to expand the training sample database of pests in other regions. In addition, the algorithm is optimized, improved, and innovated. Ultimately, it provides a new research method for intelligent plant protection and detecting crop diseases and insect pests.

## Data availability statement

The original contributions presented in the study are included in the article/supplementary material. Further inquiries can be directed to the corresponding authors.

## Author contributions

WZ designed and carried out the experimental design, selected a variety of level detection and rotation detection algorithm models for comparative analysis and research, and wrote the manuscript of the paper. The XX screening data set is annotated with horizontal and rotational labels and article grammar and image modifications. JD, XM, and ZZ proposed the overall framework design for this paper and conducted experimental research to guide it. TC participated in the experimental design and provided constructive comments. JD are the project directors. GZ provides raw pest data samples. All the authors contributed to this article and approved the submitted version.
